# Definitions of positive health: a systematic scoping review

**DOI:** 10.1177/17579759221139802

**Published:** 2023-01-10

**Authors:** Yuliya Bodryzlova, Gregory Moullec

**Affiliations:** Université de Montréal École de Santé Publique, Montréal. Canada

**Keywords:** positive health, salutogenesis, health promotion, health assets

## Abstract

An agreed definition, model, and indicators of positive health would contribute to a better understanding and wider use of the term, thus favoring the development of the positive health approach in public health. However, there is no consensus even on the definition of positive health. In this study, we systematically reviewed its definitions. We conducted a scoping review as per PRISMA guidelines. We queried the MEDLINE, Embase, PsychINFO, and Web of Science databases. The PubMed search equation was: ‘positive health’ [Title/Abstract] AND (‘health’ [MeSH] OR ‘health status’ [MeSH] OR ‘health status indicators’ [MeSH]). Definitions of positive health referring to a ‘one-dimensional’ conceptualization of health are: (i) positive health as a state ‘far beyond a mere absence of disease’; (ii) positive health as wellbeing; and those referring to a ‘two-dimensional’ conceptualization are (iii) positive health as resilience and (iv) positive health as (a reserve in) capacities. This work contributes to the refining of the salutogenic vocabulary. At this stage of the ongoing discussion on health promotion vocabulary, we propose the ‘reserve in capacities’ as the candidate for the definition of positive health.

## Introduction

The term ‘positive health’ was first proposed in the early 1950s (1) and, since then, it has been constantly rejected and reintroduced. The reasons for rejection have been the lack of consensus on its very meaning as well as doubts about the feasibility of its operationalization, while the reason for its reintroduction has been the urge researchers and practitioners have to improve health by reinforcing its sources.

The critics of the concepts underline that the current World Health Organization’s definition of health as a state of complete physical, social, and mental well-being (2) already includes positive health states, so there is no need to introduce a new concept with the same meaning (3,4). The concept of positive health itself has been criticized for being elusive, ephemeral (5), utopian (3), and misleading (6). Other critics have expressed concerns about the variability of its meanings and definitions and the difficulty of operationalizing it, no matter how it is defined (4).

On the other hand, a growing number of voices in favor of ‘making the profession of health about health’ (7) is calling for clearer definitions of positive health (4). These voices have become stronger with the publication of the theory of salutogenesis (8,9) by Antonovsky, who proposed studying the forces that enable the reproduction of health. According to the theory, the so-called ‘sense of coherence’ contributes to the better ‘reproduction’ of health (in Antonovsky: shifts the position on ‘ease-dis-ease continuum’ to the ‘ease’ end) by helping overcome the stress associated with (otherwise destructive) life challenges. The ‘general resistance resources’ (10) (wealth, status, knowledge, etc.) were called the sources of the sense of coherence. The theory was welcomed enthusiastically; however, a series of later empirical studies have shown its limits. First, a pretty modest association between the sense of coherence and health was found (11): the former mainly contributes to wellbeing, life expectancy, functional autonomy, and quality of life (12,13). Second, there is still no consensus on the list of resources that contribute to the sense of coherence. As well, the role of ecological factors, which contribute to health, is overlooked (14).

Nevertheless, these limits of the salutogenic theory in its classical form have encouraged researchers to pursue its further development (15,16). Presently, salutogenesis is viewed as an ‘umbrella term’ (15), which unites many different ‘positive’ health concepts, such as wellbeing, resilience, and quality of life; ‘positive health’ may be only one of them. It is widely used in empirical studies, and there is a body of theoretical literature on it; however, there has not been any consensus on its definition.

The theoretical literature shows two principal approaches to viewing positive health (17). First, it is viewed as a position on a one-dimensional scale where one extremity is an illness, and the other is a sought-out ‘positive health’. This conceptualization is close to the one proposed by Antonovsky, ‘ease-dis-ease continuum’, and many authors use a one-dimensional health scale to illustrate the notion of positive health (18–20), with only a few differences. For example, Catford (18) proposes three ‘illness–wellbeing’ scales for individual, environmental and socio-political indicators of positive health. Kemm (17) states that physical, mental, and social health must be considered separately while indicating the position of the individual on the health continuum. In turn, the supporters of a two-dimensional scale approach speculate that observed health should be viewed as a sum of two relatively independent processes: salutogenesis (accumulation of reserves of health) and pathogenesis (accumulation of pathology) (21–23). They pointed out that clinical manifestations of a disease in two people with the same amount of pathology might vary importantly. This difference in the clinical picture could be explained by the difference in the quantity of certain health reserves (21,22). According to this vision of positive health, health reserves have their own sources, and there should be specific mechanisms that transform them into health (15).

Besides this core difference in the conceptualization of positive health, there is a plethora of these not fundamental but still significant differences in meaning, making the reader look for the definition of positive health first when reading on the topic. Reviewing the literature that defines the term would help outline the meanings and prepare a discussion on a possible place for the term among salutogenic concepts.

In this work, we attempt to derive the meaning of the term positive health by systematically reviewing how the concept is defined/used by different authors.

## Method

We conducted a scoping review (24) as per PRISMA guidelines, an extension for scoping reviews (25). YB and GM had done a pilot search and extraction of data. The protocol for the study was discussed during several team (YB, GM) meetings. We searched for both published articles and grey literature. The search strategy included the period from 1948 (adoption of the current definition of health) to 7 July 2021. The inclusion criteria were, therefore, the presence of a definition, a model, or indicators of positive health. We consulted the following databases: MEDLINE, Embase, PsychINFO, and Web of Science, and we used the reference manager software EndNote. The PubMed search equation was: ‘positive health’ [Title/Abstract] AND (‘health’ [MeSH] OR ‘health status’ [MeSH] OR ‘health status indicators’ [MeSH]). Data were extracted by YB under the supervision of GM as per the protocol. A manual reference search was also conducted.

## Results

We screened 1813 title/abstract pairs; 147 abstracts were retained for full-text screening. Of the 147 full texts, 96 were excluded as they did not include an original definition, model, or indicators of positive health; eight articles did not meet the language inclusion criterion, and no full text was found for eight articles. The remaining 41 articles and book chapters were included for analysis in this review.

### The one-dimensional scale conceptualization of positive health

Positive health as a state ‘far beyond a mere absence of disease’ originates in the field of positive psychology/positive mental health, which studies how positive attitudes influence different facets of wellbeing. Seligman (26) speculated that positive health might be viewed as a combination of wellbeing (physical wellbeing, absence of symptoms of diseases, sense of durability, etc.), biological attributes (positive end of scales measuring biological functions), and functional perfection (positive end of laboratory test and efficient ‘person–environment’ relationships). Seligman’s definition of positive health has been widely used in empirical studies. It was found to be associated in adults with optimism (27), exercise (28), and quality of life (29,30). In studies on child and adolescent health, Seligman’s positive health was operationalized, amongst other components, as good relationships with peers, parents, and teachers (31,32). In this age group, it was associated with such general health indicators as better cardiovascular fitness, better body weight profile (31), and better sleep quality (32), and more prosocial and pro-health behavior (33). Similarly, Eriksson *et al*. (34) and Ejlertsson *et al*. (30) operationalized positive health as ‘excellent self-evaluated health’ and ‘excellent subjective health-related performance’, respectively.

### Positive health as wellbeing

Another leading conceptualization of positive health as a one-dimensional concept is that of Ryff and Singer (35). They posit that wellbeing is a state that goes ‘far beyond a mere absence of disease’, and as such, it must be considered positive health. According to them (36,37), wellbeing, contrarily to previous prevailing visions, is something more important than mere ‘positive affect over negative affect’ (38): it cannot be associated with a particular moment, but rather should ‘encompass the context of the person’s entire life’. As such, there are two types of wellbeing constituting positive health: eudemonic wellbeing as the realization of personal potential; and hedonic wellbeing as the experiences of happiness and satisfaction. Ryff and Singer (37) found that positive health, viewed this way, was associated with a wide range of indicators of the good functioning of the cardiovascular, neuroendocrine, and immune systems. Many questionnaires on positive health developed later measure positive health in terms of different facets of wellbeing. For example, the Salutogenic Wellness Promotion Scale (39) measures emotional as well as vocational, environmental, social, intellectual, physical, and spiritual wellbeing. The Salutogenic Health Indicator Scale (40) measures personal characteristics (level of energy, state of morals, etc.) and interactive function (creativity, social capacity, etc.). The Psychological Well-being scale (41,42) measures self-acceptance, environmental mastery, positive relations with others, personal growth, purpose in life, and autonomy. The questionnaire score correlates negatively with measures of general health and negative affect.

### Two-dimensional conceptualization of positive health

#### Positive health as resilience

Positive health as resilience has been viewed within the framework of the Health Assets Model (43–45). According to this vision, based on the theory of salutogenesis (46), certain internal and external factors contribute to positive health, namely, to people’s better ability to resist adversities or to a higher level of wellbeing in their absence (47). Health and social outcomes might be improved if the existing assets could be identified, the populations could be included in the construction of health as an active actor, and the most efficacious way to use these assets could be found. This reinforcement of health assets as a strategy of public health was proposed to be combined with the ‘filling deficits’ approach to maximizing a population’s health. The strength of the Health Assets Model is that it views a person in interaction with their environments, at both the community and whole-society levels (48). Similarly, other authors also highlight resilience when talking about positive health. For example, and Haase (49), Haase *et al*. (50) discuss the positive health of children and adolescents with cancer in terms of resilience. The conceptualization of positive health as resilience was used in an empirical study (28), which found its association with a lower level of C-reactive protein and better quality of sleep.

### Positive health as capacities/resources

Positive health has also been defined as capacities or resources. Huber *et al*. (51) define health as the capacity to adapt and manage yourself in the face of social, physical, and emotional challenges. They found that health had six dimensions: bodily functions, mental functions and perception, a spiritual/existential dimension, quality of life, social and societal participation, and daily functioning (52). Authors propose to use the term ‘positive health’ for this conceptualization of health to distinguish it from the biomedical ‘absence of disease’ one (52). Prinsen and Terwee (53) express some doubts about the relevance and comprehensiveness of the 46-item questionnaire developed on the basis of Huber’s model of positive health. However, Van Vliet *et al*. (54) report excellent reliability and acceptable discriminant validity of its 17-item version. Other definitions of positive health as capacity were not developed this far, but they are also worth mentioning. Forrest *et al*. (55) refer to the Institute of Medicine’s definition of health as ‘the extent to which individual children [. . .] are able or enabled to [. . .] develop and realize their potential [and] develop the capacities that allow them to interact successfully with their biological, physical, and social environments’ to term the positive health as ‘useful assets that strengthen an individual’s health’. Sen (56), in his reflection on the quality of life, emphasizes that the capabilities are its source because they enable the achievement of meaningful functioning and outcomes. Taylor *et al*. (57) suggest that some personal resources could influence health in a beneficial direction. Both these works served as a source of reflection for later studies on positive health.

### Other results

There are some definitions of positive health that were difficult to classify. For example, primary prevention was once used as a definition of positive health (58). Also, Seeman (20) defines positive health as the ‘effective personal functioning in human-system terms through the use of a concept of organismic integration’. In addition, a historical overview of the philosophic basis of the concept was found in the academic literature (59–63).

### Links between definitions

Most of the mentioned authors refer to the entire spectrum of salutogenic concepts and discuss their relationships while deriving their definition of positive health. For example, Ryff *et al*. (37) underline that positive health ‘may help keep the organism from succumbing to disease, or, when illness or adversity occurs, may help promote rapid recovery’, which clearly refers to resilience. In the same vein, Singer and Ryff (64) see positive health as wellbeing in the context of ‘resilience, recovery, primary prevention, and health promotion’. Morgan and Ziglio (46) constantly claim that individual capabilities, together with community assets, are the source of resilience and wellbeing. Haase (49) also mention resources as the source of positive health if it is defined as resilience. Harrison *et al*. (45) indicate that salutogenesis transforms assets into positive health if it is defined as resilience and wellbeing. Bartley (65) emphasizes that assets contribute to wellbeing in the absence of challenges and resilience in their presence.

## Discussion

**Figure 1.**
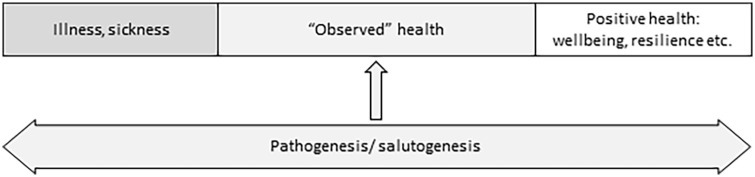
One-dimensional conceptualization of health.

**Figure 2.**
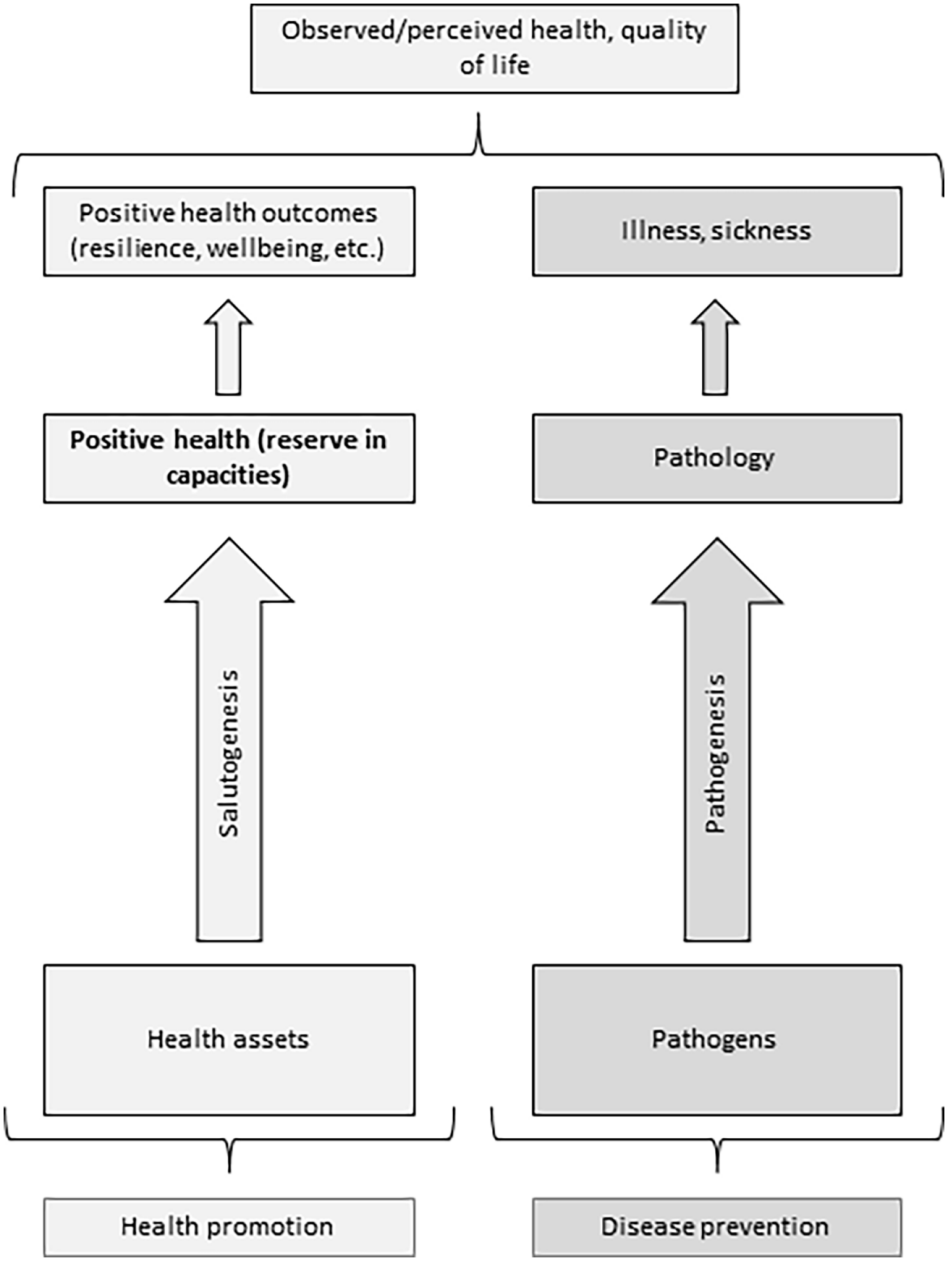
Two-dimensional conceptualization of health.

In this work, we reviewed the definitions of positive health given in theoretical and empirical studies to outline its possible meanings. The results would prepare the place for further discussion on the definition of positive health and its links with other salutogenic concepts. We found that in the framework of the ‘one-dimensional’ conceptualization of health (the current state of health is determined by the complex interplay of protective and risk factors; Figure 1), positive health is defined as a ‘state far beyond the mere absence of disease’ or as ‘wellbeing’. In the framework of the ‘two-dimensional’ conceptualization of health (the observed state of health is a sum of relatively independent pathogenic and salutogenic influences; Figure 2), positive health is defined as ‘resilience’ or ‘capacities’.

There had not been many reviews on positive health done before. Thirty years ago, Kemm (17) reviewed the conceptualization of positive health on the one- or two-dimensional scales and emphasized the limited empirical evidence for the two-dimensional model. However, today there have been some advances in the field: for example, Sperling *et al*. (66) propose viewing Alzheimer’s disease as a result of two independent influences: the pathologic brain changes on the one hand and the brain’s compensatory reserves on the other. Locker and Gibson (4) conducted a review of definitions of positive health in oral health research. They find that positive health could be defined as ‘(i) absence of negative health states; (ii) positively worded items; (iii) the positive outcomes of oral health; (iv) psychological and social attributes, and (v) the positive outcomes of chronic conditions such as oro- and craniofacial differences’. Basically, they also grouped the definitions of positive health around wellbeing (a set of psychological and social attributes, positively worded items) and around the state of an absence of diseases (the absence of negative health, positive outcomes). Our findings on the definition of positive health as the state beyond the mere absence of disease and wellbeing are close to those of Locker and Gibson; meanwhile, we added the definitions on a two-dimensional scale of health: positive health as ‘resilience’ and ‘capacities’.

While health promotion needs a more precise vocabulary (17), the discussion on the meaning of the term ‘positive health’ should continue to arrive at a definition that would satisfy the needs of researchers and practitioners in health promotion. However, the definition will depend on the accepted one- or two-dimensional conceptualization of health.

If a one-dimensional conceptualization of health is accepted, there could be two possible definitions of positive health: positive health as the state ‘far beyond the mere absence of disease’, or positive health as ‘wellbeing’. In this case, the doubts regarding the utility of positive health as a concept seem justified. The state ‘far beyond the mere absence of disease’ is unclear in both how far ‘beyond’ and far beyond what ‘disease’ the positive health should be. Defined this way, the term positive health is ‘elusive’, ‘misleading’, and even ‘demagogical’, as already stated by critics (3,4). The definition of positive health as wellbeing might also not be useful in health promotion. First, certain most valuable contributors to subjective wellbeing (love, intimacy, self-actualization) are the products of deeply intimate, lifelong personal development (67) and could not be achieved by public health interventions. Second, the pursuit of, for example, hedonistic individual wellbeing might not be a prosocial or pro-health enterprise: it might lead to unhealthy life choices in food, leisure, and substance use (68). Finally, some discomfort (lack of wellbeing) might be a natural and even desirable human reaction in the face of significant ethical or professional challenges. Nevertheless, it would be correct to unite the ‘states far beyond the mere absence of disease’ and ‘wellbeing’ under the term ‘positive health outcomes’ (69,70), which is already widely used, but to our best knowledge, also barely discussed. Positive health outcomes might be considered manifestations of the salutogenic process or ‘symptoms’ of positive health (17).

On the other hand, if we accept that observed or actual health is formed by two parallel and relatively independent processes, that is, salutogenic and pathogenic, positive health could be defined as ‘resilience’ or as ‘capacities’. There are three reasons we would prefer capacities over resilience as a definition of positive health. First, the very definition of resilience (resistance in case of adversities) implies the presence of adversities. Resilience as positive health might cause difficulties in the assessment of positive health in the absence of adversities, while the presence/absence of capacities might be measured under any conditions. Second, capacities might be a more specific term: for example, capacity to make a healthy food choice, capacity to exercise regularly, or capacity to engage in community activities. From this point of view, the more precise term for positive health might be some reserve in capacities. The reserve in capacities would facilitate more rapid and flexible adaptation, more efficient use of assets, and manifest in higher wellbeing, resilience, and other possible positive health outcomes. Third, Forrest *et al*. (55), defining positive health as useful assets, emphasize that ‘[A] ssets can be biological [. . .], functional [. . .], behavioral [. . .], or experiential’. Similarly, the definition of positive health as a reserve in biological, emotional, behavioral, or social/societal capacities enables the fixing of tangible and intuitively clear objectives of health promotion interventions (see Figure 2).

### Limits

Our work has several limits. First, it does not provide an entire picture of the reflections on positive health and its conceptualization: this long history that could not be reduced to the size of a single journal publication. Second, many salutogenic concepts used in the literature on health promotion (e.g. empowerment, sense of coherence, learned forcefulness) were not included in the analysis, as they were not mentioned in any direct link with positive health. Finally, the review of links between some salutogenic concepts was not as developed as needed; the complexity of their mutual relationships was simplified.

## Conclusion

Our work contributes to refining the salutogenic vocabulary before reaching a consensus on the meaning of the term positive health, which could be defined as a reserve in capacities contributing to wellbeing and resilience. Health assets are the source of positive health. A more nuanced vocabulary might enhance the salutogenic approach to health promotion.
